# Examining the informal urban growth trends in a Port city

**DOI:** 10.1016/j.heliyon.2023.e22581

**Published:** 2023-11-28

**Authors:** Cai Li, Sania Khan, Noman Sahito, Muhammad Yousif Mangi, Wadi B. Alonazi

**Affiliations:** a School of Management, Jiangsu University, Zhenjiang 212013, China; b Department of Human Resource Management, College of Business Administration, Prince Sattam Bin Abdulaziz University, Al Kharj 11942, Saudi Arabia; c Department of City & Regional Planning, Mehran University of Engineering & Technology, Jamshoro Pakistan; d Health Administration Department, College of Business Administration, King Saud University, Riyadh 11587, Saudi Arabia

**Keywords:** urban growth, Geographic information system, Land change modeler, Land use land cover, Metropolitan karachi

## Abstract

Rapid urban developmental growth is a heated debate worldwide due to environmental challenges. This research has examined the spatiotemporal trend of informal built-up growth in Karachi city. Using a geo-information system, the past twenty years (2000–2020) trends of informal built-up growth are examined. For attaining the research objectives, geo-referenced high-resolution maps and satellite images are used for accuracy based spatial data. Karachi is divided into five different land use and land cover (LULC): formal built-up, informal built-up, vacant, water bodies, and green spaces. Spatial data of informal built-up growth change of five different years, 2000, 2005, 2010, 2015, and 2020 are generated through acquired maps digitization using ArcMap. Subsequently, the gains and transfers of Karachi's informal built-up growth based on five years 2000–2005, 2005–2010, 2010–2015, and 2015–2020 are analyzed using the Land Change Modeler (LCM) in IDRISI software. Also, land use land cover changes (LULCC) are predicted for the next 40 years (2020–2060) using the integrated Cellular Automata Markov (CA-Markov) simulation model in IDRISI. The results revealed that Karachi's built-up is expanding rapidly. Land conversion into the informal built-up area is alarming, as it has changed from 144.31 km^2^ to 217.19 km^2^ with 72.88 km^2^ in the past twenty years (2000–2020) and has occupied green and agricultural land. Most informal built-up areas have transitioned from vacant (71.01 km^2^) land use land cover (LULC). The informal built-up area could expand from 217.19 km^2^ to 317.63 km^2^, with about 100.44 km^2^ up to 2060. The planned and unplanned development will be towards the city's East (E) direction and will convert and ruin agriculture and vacant land. The present study provides suggestions to urban planners, administrative authorities, and policymakers to control informal growth and achieve sustainable development goals in developing countries.

## 1 Introduction

In recent decades, urbanization has been increasing internationally, which may reach up to 70 % by 2050 [1,2]. It is the practice of people to migrate to urban areas for a better lifestyle [3,4]. The rural-urban migration is accelerating daily [5,6]. Consequently, enormous physical, socioeconomic, and environmental effects increase continuously [7,8]. The transformation of the world is happening due to rapid increases in urban population, the physical expansion of cities [7], the high population density, huge concrete structures, large impervious surfaces, alarming climatic, environmental and hydrological situations, increasing demand for transportation and emission of CO_2_ [9,10]. As a result, urban areas are facing many severe problems: poor public services, traffic jams, insufficient housing, poor education, bad health conditions, and increasing demand for employment [1,2,11,12], creation of slums, safety and security, demand of energy and clean water [[13], [14], [15], [16]], municipal solid waste management, and so on [17]. With rapid urbanization, these problems emerge and affect the urban life [12,18].Moreover, the rapid trend of urbanization and physical growth of cities have dramatically transformed the land use land cover (LULC) (e.g., built-up patterns) in areas, particularly in developing countries [19,20]. Karachi, Pakistan, also suffers [[21], [22], [23]]. These urban areas have been experiencing considerable growth in recent decades [20,21,24].

Pakistan's urbanization and physical expansion of cities are not new; they have been undergoing since 1947, after the independence. However, they have rapidly mounted since 1971 [25,26]. According to a recent census report, Karachi has 36.4 % of the total population of the country [27]; by 2050, the population may reach 50 % [1,27,28]. With the increasing population, Karachi is facing multiple issues: natural, socioeconomic, physical, and development control circumstances [6,[29], [30], [31], [32], [33]].

The problems of Karachi, Pakistan, are the main obstacles to sustainable development and an environment where planning and monitoring are not implemented appropriately [[34], [35], [36]]. The area contains suburbs recently counted as the nineteenth most populated city in the world [23,37]. Karachi's built-up land use land cover (LULC) is also expanding rapidly. In the last fifty-five years (1955–2010), cities built up land use land cover (LULC) have expanded to 715.794 km^2^ with an average 13.35 % annual growth ratio [38]. Urbanization converts agriculture and barren land into informal growth [38,39]. Increasing rapid urbanization creates informal growth in the city; currently, more than 50 % of the city is informally built [[40], [41], [42]].

## 2 Literature review

Land use land cover change (LULCC) is a key factor in improper urban built-up or growth transitions [19,43,44]. Therefore, spatial patterns and temporal variation knowledge related to land use land cover (LULC) is essential to understanding the dynamics of the physical growth of urban areas associated with built-up change [[45], [46], [47]]. Recognizing land use/land cover change consistently has been important for planning and managing urban built-up and its expansion [44,48,49]. Technological-based geo-information techniques have been widely employed by scholars internationally for identifying and monitoring the changes that occurred in land use land cover (LULC) over time to manage urban growth efficiently [48,[50], [51], [52], [53]].

Worldwide, academia has mainly employed a variety of automatic land classification approaches in their studies to extract the timely spatial data of land use land cover change (LULCC) considering remote sensing (i.e., Landsat, etc.) images, such as; maximum likelihood classification (MLC) [49,54,[55], [56], [57], [58],^59^,^60^], minimum distance classification (10.13039/100005637MDC) [61,62], random forest (RF) [24,50,63], support vector machine (SVM) [[64], [65], [66]], multi-layer perception neural network (MLP NN) [62,67], and object-oriented classification [[68], [69], [70]] methods, etc.

However, remotely sensed imagery data are not detected accurately due to less visibility of earth surfaces (i.e., a maximum of 30X30 each pixel size-based data can be extracted from Landsat images). Therefore, to have the highest accuracy in land use land cover (LULC) spatial data, the geo-referenced based high Resolution of previous governmental detailed maps and authorized satellite images [71] (referred to in table 3.1) of different interval years of Karachi by responsible authority (http://www.kmc.gos.pk/) were used in this research. Simultaneously, different geometric-based scientific models have been widely applied in several studies to assess the land use/land cover transformation internationally, such as the multi-agent System [72,73], Cellular Automata (CA) Model [74,75], Markov chain analysis (also called transition matrix) [63,76,77], Expert Models [[78], [79], [80], [81]], Land Change Modeler (LCM) [82], Logistic Regression [[83], [84], [85]], and Evolutionary Models [[86], [87], [88]], etc. However, an integrated Cellular Automata Markov (CA-Markov) Model [19,68,[89], [90], [91], [92]] has been preferred by academia while conducting land use land cover (LULC) studies around the world due to virtue of its spatiotemporal attributes and perfect predictions [19,92].

For instance, Liping, Yujun and Saeed [93] explored the land use land cover changes (LULCC) and predicted them by employing the CA-Markov Model in the Jiangle hilly area of China. Xiong, Beckmann and Tan [89] studied land use land cover changes (LULCC) that occurred through extensive construction of the Airport's infrastructure by applying the CA-Markov method in Hangzhou, China. Nguyen et al. [92] used Cellular Automata-Markov modelling to evaluate the urban landscape patterns between 1990 and 2030 in Hanoi City, Vietnam. Similarly, Abdullahi and Pradhan [91] undertook CA-Markov as a land use land cover change (LULCC) model considering urban density, mixed-use development, and intensity parameters and evaluated future urban expansion and land use land cover change (LULCC) in Kajang City, Malaysia.

Likewise, Wang, Derdouri and Murayama [94] simulated spatial patterns and temporal variation of land use land cover change (LULCC) situations through CA-Markov Model in the Tokyo Metropolitan, Japan, as well; Zadbagher, Becek and Berberoglu [68] carried out a study on land use land cover change (LULCC) at the regional scale and predicted land use land cover change (LULCC) transformation by adopting Cellular Automata-Markov modular in Seyhan Basin, Turkey.

Whereas the Markov chain predicts the land use land cover change (LULCC) at the time t+1 based on land use land cover (LULC) structure quantity at the time t using conversion probabilities matrix extracted from prior land use land cover (LULC) transitions between each land use land cover (LULC) type [51,63,68,76,90]. Markov technique is often suitable for predicting the land use land cover change (LULCC). Still, it does not have adequate capacity to represent the spatial conditions as the Markov chain ignores the change in spatial patterns [43,91,92,95]. At the same time, Cellular Automata (CA) is a dynamic and synchronous mechanism where the state of cells and their neighborhood conditions at the present point in time decide their form at the next moment [74,89,91]. Cellular Automata fundamentally detect the change in the spatial location [75,85,90]. The Cellular Automata is capable and can deal with the evolution of spatiotemporal dynamics within complex systems of spatial distribution [74,89].

Therefore, integrating Cellular Automata and Markov as a CA-Markov model is recognized as a more powerful and useful technique to simulate the spatiotemporal dynamics change. CA-Markov approach is highly applicable for predicting the land use land cover change (LULCC) situations, particularly in rapidly urbanizing areas, and also identifying the periodic transition state of different land use/land cover [67,68,89,91,92,59,60]. Lastly, accuracy assessment (i.e., producers' accuracy, user accuracy, overall accuracy, and Kappa Coefficient) could be undertaken using satellite imagery software to confirm the values between classified land use land cover (LULC) results and ground reality [24,62,96]. Thus, researchers are getting suitable periodic land use land cover change (LULCC) results.

However, informal (or unplanned) urban built-up growth-based research is conducted. The most urbanized and rapidly developing city, Karachi, Pakistan, is selected as the study area. Satellite imagery information based on urban built-up growth situations in the past two decades (2000–2020) with five-year intervals is assessed and predicted up to the next 40 years (2020–2060) in Karachi. This research has specifically pointed out the proportion of urban land transformation into informal (or unplanned) built-up growth in Karachi, Pakistan.

## 3 Material and methods

### 3.1 Study area

**Fig. 1:**
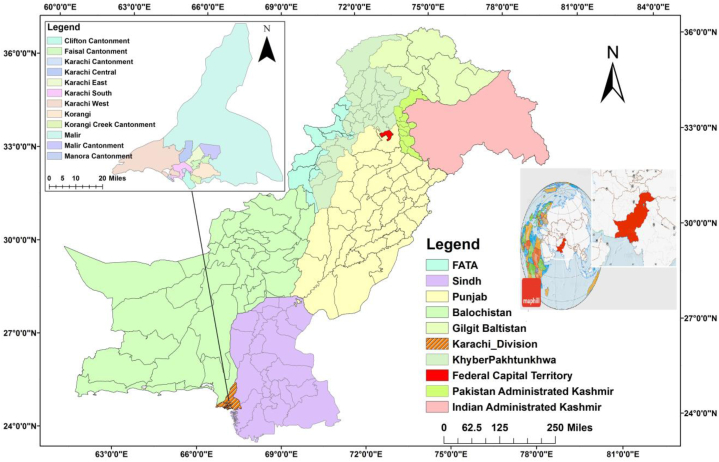
Location of Karachi, Pakistan [100].

Karachi is situated in the southern part of Pakistan on the Arabian Sea (Fig. 1) [41], the Capital of Sindh Province and Pakistan's largest and most cosmopolitan city [97]. Karachi's boundaries are spread over approximately 3600 km^2^ [98]. The city has six districts: “Karachi East, Karachi West, Karachi Central, Karachi South, Korangi, and Malir, governed under the Metropolitan Corporation and City District Government Karachi” [41,99]. The city has 62 % of the total province population per the 2017 census. Karachi is the country's major Seaport and is Pakistan's significant financial hub [97].

### 3.2 Methods

This research analyzed the spatiotemporal trend of informal urban built-up growth in the rapidly developing Karachi, Pakistan. A detailed research framework is designed to achieve the research objectives [2,101] to collect and analyze the spatial data. The different methods of spatial data collection and analysis are widely discussed as follows.

### 3.3 Spatial data collection and analysis

The trend of Karachi's informal (or unplanned) built-up growth that happened since the past two decades (2000–2020) was assessed with five years of the interval through a systematic land use land cover (LULC) classification and predicted informal (or unplanned) urban built-up growth scenarios up to next 40 years (2060) by employing geo-information system tools in both ArcGIS10.8 and IDRISI 17.0 software.

However, having the higher accuracy in spatial data, the geo-referenced based high-resolution, detailed maps and satellite images [71] of different years of Karachi were taken from governmental departments (http://www.kmc.gos.pk/) in tiff format. The information about various spatial data collection sources, such as high-resolution maps and satellite images, is provided in Table 1.

**Table 1:** Spatial data collection sources.

Year	Type of Image Format	No. of Bands	Spatial Resolution (m)	XY Coordinate System
2000	Satellite Image (Tiff)	9	14.25, 14.25	WGS_1984_UTM_Zone_42 N
2005	Geo-referenced Map (Tiff)	3	15.98, 15.98	WGS_1984_Web_Mercator_Auxiliary_Sphere
2010	Satellite Image (Tiff)	3	0.88, 0.88	WGS_1984_UTM_zone_42 N
2015	Satellite Image (Tiff)	3	2.54, 2.54	GCS_WGS_1984
2018	Geo-referenced Map (Tiff)	4	4.34, 4.34	WGS_1984_Web_Mercator_Auxiliary_Sphere

Apart from this, considering the Pakistan land classification system concerning Karachi [102], land use land cover (LULC) types were segregated into four categories: Urban/Built-up [24,103], Vegetation/Green Spaces [104,105], Barren Land/Vacant [104,106], and Water Bodies [107,108]. While achieving the goal of this research, the Urban/Built-up category was further subdivided into formal and informal built-up land use land cover (LULC), as briefly elaborated in Table 2.

**Table 2:** Delineation of land use/land cover categories based on spatial data classification.

Category	Sub-category	Description
Urban/Built-up		Residential, industrial areas, commercial activities, communication, transportation, playgrounds, construction materials, mixed urban settlements, and other urban uses [24,103,109]
	Formal Built-up	Planned/proper urban developed and under-developing settlements [42]
	Informal Built-up	Sprawl/spontaneous/illegal/unplanned development, e.g., slums, katchi Abadie's, squatter settlements, leap-frog/disperse settlements [110]
Barren Land/Vacant		Stones and boulders areas, gravels, bare rock areas, hardpan and bare soil areas, and other open areas [104,106]
Water Bodies		Permanent open water, reservoirs, streams, lakes, bays and estuaries, and other water bodies [107,108]
Vegetation/Green Spaces		Agriculture areas, pasture, plowed areas, golf courses and parks, and other vegetation [104,105,109]

**Fig. A1:**
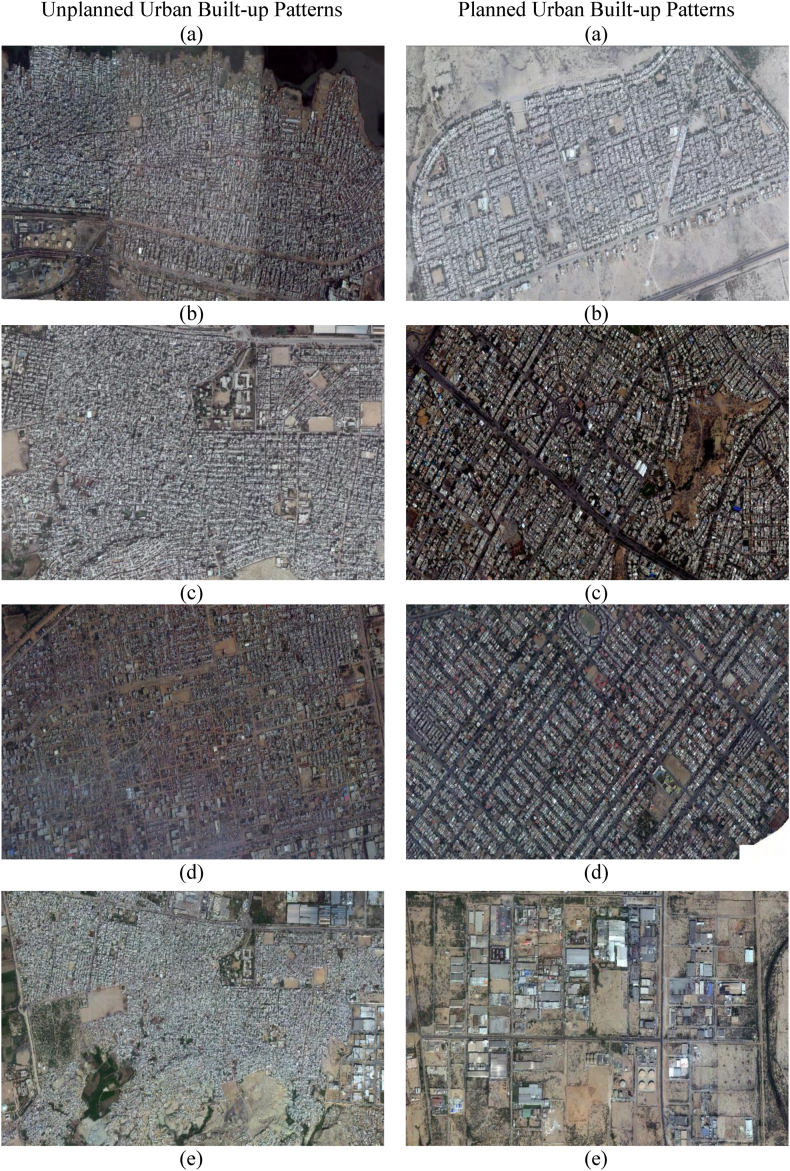

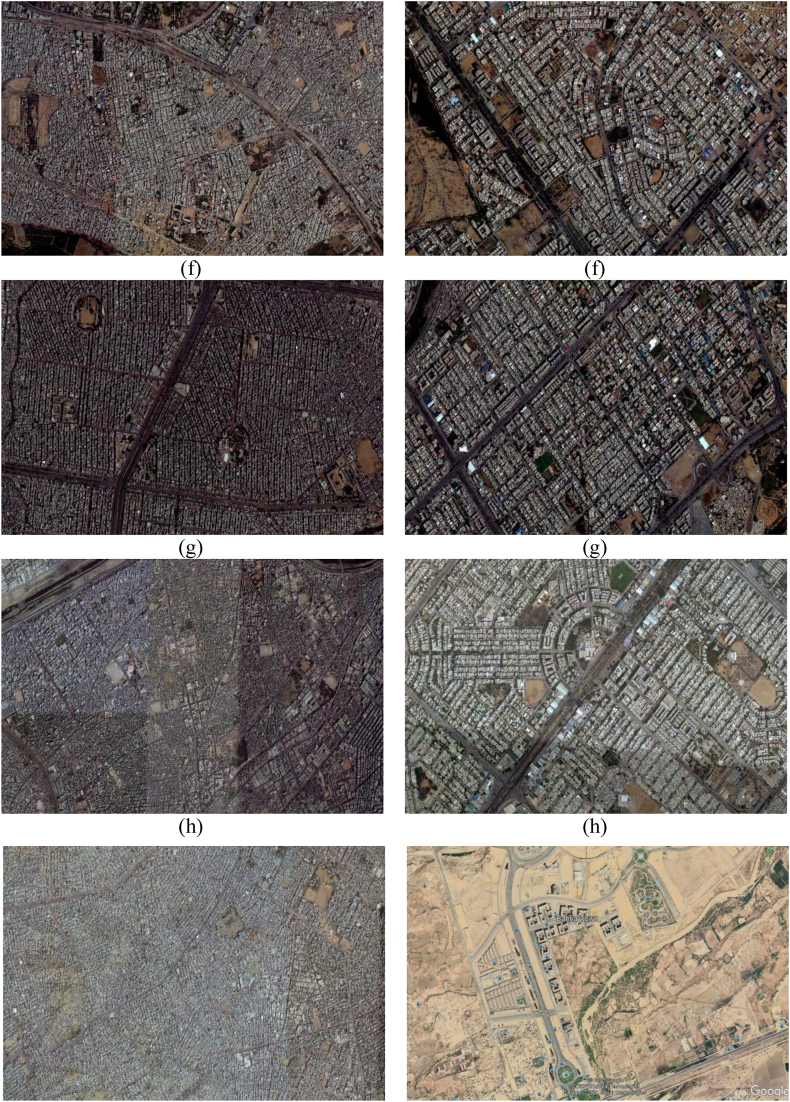
Various urban built-up patterns of Karachi (Google Earth Pro).

The spatial data of built-up (i.e., formal and informal built-up) growth transitions of five different years (i.e., 2000, 2005, 2010, 2015, and 2020) were then generated by digitizing acquired maps using ArcGIS10.8. Google Earth Pro, as shown in Figure A1.

Subsequently, digitized spatial data attributes were re-coded [62,109,111,112], segregating into five land use land cover (LULC) classes: formal built-up, informal built-up, vacant, water bodies, and green space. These classes were then converted into a raster dataset individually. All raster layers were adjoined employing data management (mosaic to new raster) tool and formed different land use land cover (LULC) classified maps by raster dataset of data management tools in ArcMAP10.8. Afterwards, classified raster datasets were also converted into ArcInfo raster ASCII format for exporting the GIS data to IDRISI 17.0 software data format.

Many researchers in different studies the geo-informatics software has used to simulate land use land cover (LULC) or urban growth change [19,54,75,113,114]; however, they all are complex software for land use land cover (LULC) or Urban Growth Modeling. IDRISI Selva [82,85,114,59,60] is a user-friendly software for analyzing spatiotemporal land use land cover (i.e., urban built-up growth) change (LULCC) data and predict them up to desirable period. Hence, the Land Change Simulation Modeler (LCM) [82,114] was adopted and performed change analysis technique identifying the gains and losses of different land use land cover (LULC), particularly urban built-up (i.e., informal built-up) growth between two specified periods. Four spatiotemporal land use land cover change (LULCC) maps were prepared based on five-year intervals from 2000 to 2020 (i.e., 2000–2005, 2005–2010, 2010–2015, and 2015–2020). They examined the urban built-up (i.e., informal built-up) growth situations.

### 3.4 Accuracy assessment of classified land use land cover (LULC) or urban built-up growth maps

At the same time, spatiotemporal land use land cover (LULC) maps' accuracy assessment was undertaken considering "K" (i.e., K_no_, K_location_, K_locationStrata_, and overall Kappa K_standard_) scores [115,59,60] to validate the values between classified land use land cover (LULC) results and ground reality [24,62,96] by employing GIS analysis based change/time series' validate tool in IDRISI software. Thus, periodic (2000–2020) land use land cover change (LULCC) results were acquired appropriately.

The overall Kappa (K_standard_) value of all classified land use land cover (LULC) or urban built-up growth maps exceeded 70 % (or 0.7). Hence, all maps' land use land cover (LULC) or built-up growth change processes performed strongly and accurately [115]. Whereas, all remaining "K" values (K_no_, K_location_, and K_locationStrata_) of various land use land cover (LULC) or urban built-up growth maps also surpassed 85 %, as almost no errors or minor quantification and location errors were found between the different periods' land use land cover (LULC) maps in Karachi [115].

### 3.5 Informal urban built-up growth prediction

Afterwards, informal (or unplanned) urban built-up growth was predicted for the next 40 years up to 2060 with ten years of interval (i.e., 2030, 2040, 2050, and 2060). To do so, firstly, four various time interval-based transition probability matrixes were produced using the Markov Model [76,116] by putting transition time periods (20 years) between earlier (2000) and later (2020) land use land cover (LULC) spatial data maps and forward projecting time periods from the later (2020) map (i.e., 10, 20, 30 and 40 years) in IDRISI. However, urban built-up (i.e., informal/unplanned built-up) growth transition's mathematical equation process to which Markov-based transition probability matrixes of different time periods were generated as expressed below:

The limited initial-order Markov procedure is a random method concerning the property, which value X_t_ at the time t only depends on the value X_t–1_ at the time t–1. It is not associated with the values of X_t–2_, …, X_0_ [77,92,116] as indicated in equation (1).

(1) $$P\left\{X_{t}=a_{j}|X_{0}=a_{0},X_{1}=a_{1},\ldots X_{t–1}=a_{i}\right\}=P\left\{X_{t}=a_{j}|X_{t–1}=a_{i}\right\}$$

Where t = 0, 1, 2 …

The probability of urban built-up (i.e., informal/unplanned built-up) growth change from time period a_i_ to a_j_ is a one-step change probability as P {X_t_ = a_j_|X_t–1_ = a_i_}. However, whenever a homogeneous Markov chain [77,116] is undertaken, the transition probability also considered equation (2) as illustrated as follows:

(2) $$P\left\{X_{t}=a_{j}|X_{t–1}=a_{i}\right\}=P_{ij}$$

Hence, the transition probability was assessed through the statistical formula given below:

(3) $$P_{ij}=\frac{n_{ij}}{n_{i}}$$

where n_ij_ is the number of times that the urban built-up (i.e., informal/unplanned built-up) growth transitioned from condition i to j, and n_i_ is the number of times that urban built-up (i.e., informal/unplanned built-up) type a_i_ happened [77,116].

Considering all transition probabilities among all situations, a transition matrix was proposed as portrayed as follows:

(4) $$P=\left(\begin{array}{l} \begin{array}{c} p_{11}p_{12}\ldots p_{1m}\\ p_{21}p_{22}\ldots p_{2m}\\ \vdots \vdots \ddots \vdots \\ p_{m1}p_{m2}\cdots p_{mn} \end{array}\\ ∙ \end{array}\right)$$

Therefore, the transition probability matrix in "n" steps was easily identified by the following formula [77,116]:

(5) $$P^{\left(n\right)}=P^{n}$$

**Fig. 2:**
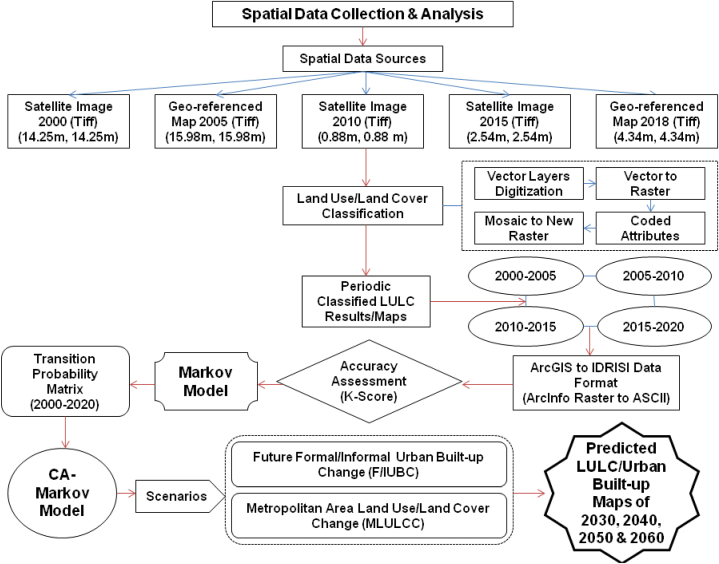
Research method framework.

More significantly, the analysis process of the Markov model is limited while performing the land use/land cover (or spatial distribution of LULC) simulation using scientific methods [91,92]. Therefore, in the end, to overcome this limitation, CA-Markov as integrated Cellular Automata and Markov simulation model [89,91,92,94] was employed and predicted the future informal (or unplanned) urban built-up growth trend of Karachi up to 2060 into four phases (i.e., 2030, 2040, 2050, and 2060) using IDRISI software. CA-Markov is a suitable and unbiased scientific approach to predicting the rate of change in land use land cover change (LULCC) [57,89,90]. The methodological framework for spatial data collection and analysis is illustrated in Fig. 2.

## 4 Results

Urban built-up (planned and unplanned) classifications change statistics are identified based on five years of interval (i.e., 2000–2005, 2005–2010, 2010–2015, and 2015–2020) between 2000 and 2020 in Karachi. The total urban built-up area was assessed as 498.03 km^2^, 538.44 km^2^, 595.92 km^2^, 667.01 km^2^, and 749.78 km^2^ after every five years of intervals in 2000, 2005, 2010, 2015, and 2020 respectively. However, the formal (or planned) built-up area was extracted at 353.72 km^2^ (71.02 %), and informal (or unplanned) built-up was 144.31 km^2^ (28.98 %) in 2000. The formal (or planned) built-up area was observed at 376.93 km^2^ (70 %), and informal (or unplanned) built-up was 161.51 km^2^ (30 %) in 2005 after five years of interval.

Similarly, in 2010, the formal built-up area was spread over 410.41 km^2^ (68.87 %), and the informal built-up area was 185.51 km^2^ (31.13 %). Whereas formal (or planned) built-up land use land cover (LULC) was noted at 466.34 km^2^ (69.91 %), and informal (or unplanned) built-up was 200.67 km^2^ (30.09 %) between 2010 and 2015. Besides, in the past five years (2015–2020), the total urban built-up classifications change statistics were taken at 749.78 km^2^, in which the planned built-up area was laid at 532.59 km^2^ (71.03 %). The unplanned built-up area was 217.19 km^2^ (28.97 %) in Karachi, as briefly discussed in Table 3.

**Table 3:** Urban built-up classifications change statistics of Karachi (2000–2020).

Built-up Classes	2000		2005		2010		2015		2020	
	Area (km^2^)	Ag%	Area (km^2^)	Age %	Area (km^2^)	%	Area (km^2^)	%	Area (km^2^)	%
Planned/Formal Built-up	353.72	71.02	376.93	70.00	410.41	68.87	466.34	69.91	532.59	71.03
Unplanned/Informal Built-up	144.31	28.98	161.51	30.00	185.51	31.13	200.67	30.09	217.19	28.97
Total Built-up Area (km^2^)	498.03	100	538.44	100	595.92	100	667.01	100	749.78	100

**Fig. 3:**
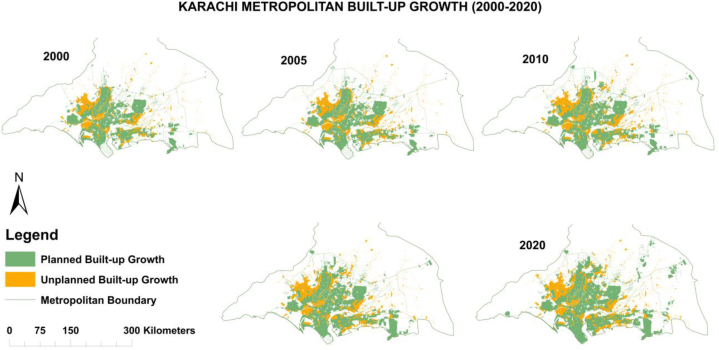
Urban built-up classification maps from 2000 to 2020 in Karachi.

**Fig. 4:**
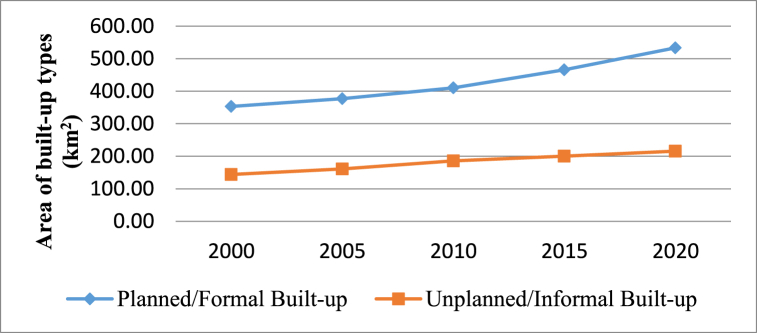
Urban built-up change trends from 2000 to 2020 in Karachi.

Urban built-up considering formal (or planned) and informal (or unplanned) built-up growth change statistics revealed that both formal and informal built-up growth have changed with time. Formal and informal urban built-up growth scenarios based on five-year interval maps between 2000 and 2020 are given in Fig. 3 and the trend the trend in Fig. 4.

### 4.1 Urban built-up growth change data validation/accuracy assessment

**Fig. 5:**
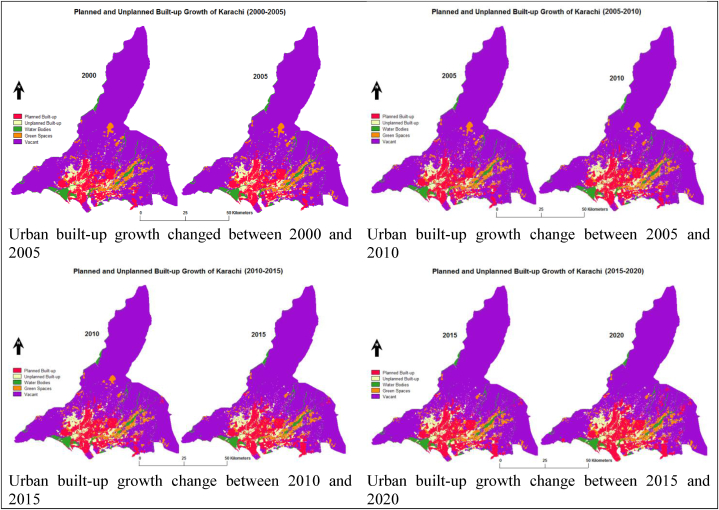
Urban Build-up growth changes between 2000, 2005, 2015 and 2020.

The spatial patterns and temporal variation results were validated using urban built-up (planned and unplanned) growth change maps. They investigated the data accuracy through "K" scores using the change/time series validation tool of GIS analysis in IDRISI Selva 17.0. The urban built-up (planned and unplanned) growth change maps of different time intervals as 2000–2005, 2005–2010, 2010–2015, and 2015–2020 were validated, inquiring about the maximum accuracy in spatial data between two classified land use land cover (LULC) maps as indicated in Fig. 5.

Urban built-up growth maps were calculated, including formal and informal built-up uses "K" scores between 2000 and 2020 based on five years of intervals. K_no_ was noted at 0.9987, K_location_ was 0.9987, K_locationStrata_ was 0.9925, and the overall Kappa (K_standard_) was observed at 0.9883 between 2000 and 2005 urban built-up growth maps. Concurrently, spatiotemporal data between 2005 and 2010 urban built-up growth maps were validated with "k" scores as K_no_ was 0.9951, K_location_ was 0.9951, K_locationStrata_ was 0.9898, and overall Kappa (K_standard_) was assessed as 0.9840. Meanwhile, K_no_ was 0.9907, K_location_ was 0.9907, K_locationStrata_ was 0.9863, and overall K_standard_ was 0.9787 between 2010 and 2015 built-up growth maps.

Also, more recently passed spatiotemporal interval from 2015 to 2020 urban built-up (planned and unplanned) land use land cover (LULC) data accuracy was measured by Kappa scores. Hence, K_no_ was calculated at 0.9960, K_location_ was 0.9960, K_locationStrata_ was 0.9865, and the overall Kappa (K_standard_) score was observed at 0.9791 between 2015 and 2020 spatial data. Additionally, accumulative 20 years of interval-based spatiotemporal data were also validated. The overall Kstandard value was taken at 0.9354, and the remaining "K" scores were 0.9586 (K_no_), 0.9812 (K_location_), and 0.9812 (K_locationStrata_), respectively, between 2000 and 2020, as shown in Table 4 and Fig. 5.

**Table 4:** Accuracy assessment/Validation of built-up growth maps ("K" scores).

K Indicators (Geo-referenced Spatial Data Images)	K_standard_	K_no_	K_location_	K_location Strata_
2000–2020	0.9354	0.9586	0.9812	0.9812
2000–2005	0.9883	0.9987	0.9987	0.9925
2005–2010	0.9840	0.9951	0.9951	0.9898
2010–2015	0.9787	0.9907	0.9907	0.9863
2015–2020	0.9791	0.9960	0.9960	0.9865

Urban built-up growth change's data accuracy outcomes revealed that the overall Kappa (K_standard_) value of all built-up growth maps segregating into formal and informal built-up land use land/cover change (0.9883, 0.9840, 0.9787, and 0.9791) exceeds 70 % (or 0.7). Hence, land use land cover change (LULCC) models of all built-up (planned and unplanned built-up) growth maps have performed very strongly and accurately. Whereas the remaining "K" values (K_no_, K_location_, and K_locationStrata_) of urban built-up (planned and unplanned) growth change maps exceed 85 %, which means that almost no errors or minor quantification and location errors were found between the different urban built-up growth change maps in Karachi [115].

### 4.2 Gains and transfers in formal (planned) and informal (unplanned) urban built-up

The increases and losses in formal and informal urban built-up land use land cover (LULC) classes are examined using the Land Change Modeler (LCM) in EDRISI software. The land change analysis tool is performed in LCM by putting different time periods based on urban built-up growth change maps (parameters) the past 20 years (i.e., 2000–2005, 2005–2010, 2010–2015, 2015–2020, and 2000–2020) for extracting the gains and losses rate (i.e., km^2^ per year) of both planned and unplanned built-up uses. Gains and transfer rates of each time period are analyzed individually.

However, 251.75 km^2^ of urban built-up areas have gained from other land use/land cover (i.e., vacant, water bodies, and green spaces) and fewer transferred (−3.8 km^2^) between 2000 and 2020 (20 years interval). The total formal built-up has gained 178.87 km^2^ and lost −2.29 km^2^ area with an average of 8.94 km^2^ gains and −0.12 km^2^ losses per year. Whereas total informal (or unplanned) built-up has gained 72.88 km^2^ and transferred −1.51 km^2^ area with 3.64 km^2^ gains and −0.08 km^2^ losses average per year as clarified in Table 5 and Fig. 7.

**Table 5:** Gains and losses of urban built-up (planned and unplanned) in different years (2000–2020).

				Losses		Gains	
Year	LULC Classes	Persistence/Unchanged Area	%	Area (km^2^)	Average Losses/Year (km^2^)	Area (km^2^)	Average Gains/year (km^2^)
2000 & 2005	Planned/Formal Built-up	376.93	70.00	−0.077	−0.015	23.21	4.642
	Unplanned/Informal Built-up	161.51	30.00	−0.033	−0.007	17.2	3.44
	Total Built-up Area (km2)	538.44	100	−0.11	−0.022	40.41	8.082
2005 & 20010	Planned/Formal Built-up	410.41	68.87	−0.110	−0.022	33.48	6.696
	Unplanned/Informal Built-up	185.51	31.13	−0.050	−0.01	24	4.80
	Total Built-up Area (km2)	595.92	100	−0.160	−0.032	57.48	11.496
	
	Planned/Formal Built-up	466.34	69.91	−1.056	−0.211	55.93	11.186
2010 & 2015	Unplanned/Informal Built-up	200.67	30.09	−0.454	−0.091	15.16	3.032
	Total Built-up Area (km2)	667.01	100	−1.51	−0.302	71.09	14.218
2015 & 2020							
	Planned/Formal Built-up	533.59	71.17	−1.822	−0.364	66.25	13.25
	Unplanned/Informal Built-up	216.18	28.83	−0.738	−0.147	16.51	3.302
	Total Built-up Area (km2)	749.78	100	−2.56	−0.512	82.76	16.552

**Fig. 6(a):**
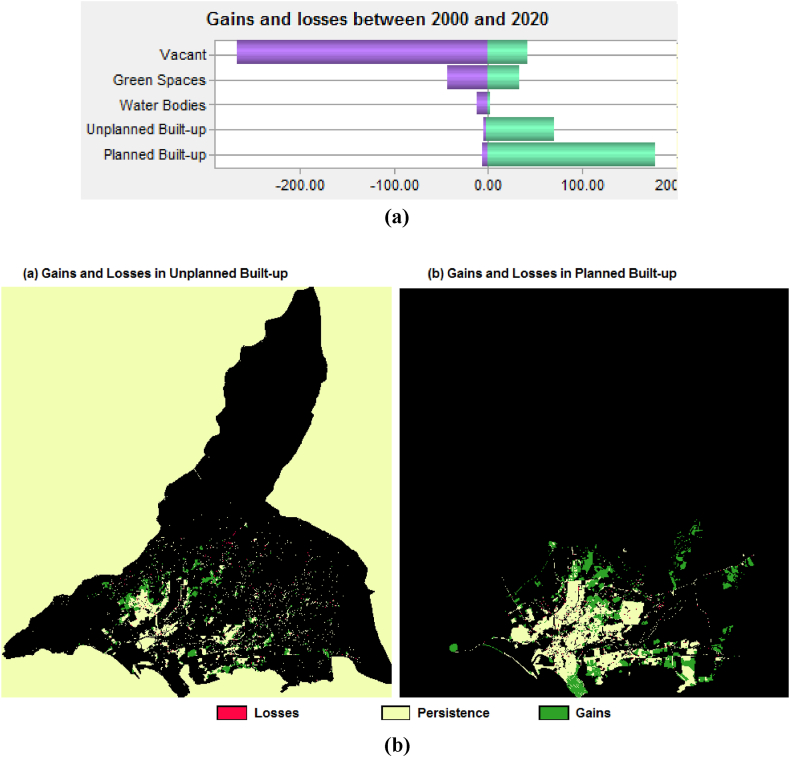
Gains and losses of urban built-up between 2000 and Fig. 6(b). Gains and losses situation of planned and unplanned built-up from 2000 to 2020.

**Fig. 7:**
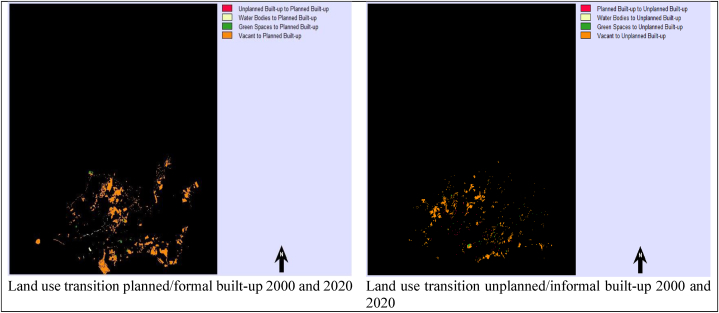
LULC formal and informal urban built-up areas 2000 and 2020.

In addition, the gains and losses situations of planned and unplanned built-up areas between 2000 and 2020 are drawn in Fig. 6(a) and (b) and shown in Fig. 7. The red represents the built-up (planned and unplanned) losses in the specified 20 years of tenure, whereas the green represents the gains in formal and informal built-up areas. The remaining half-white colour indicates the persistence or unchanged built-up areas.

### 4.3 Land use land cover (LULC) transitions into formal and informal urban built-up

Urban built-up areas considering formal (or planned) and informal (or unplanned) built-up changes from other land use land covers (LULC) are checked for the past 20 years between 2000 and 2020. The area is divided into five different land use land covers (LULC): planned built-up, unplanned built-up, water bodies, green spaces, and vacant areas. Subsequently, the land use transition process was performed using the Land Change Modeler in IDRISI software.

According to results, a total of 178.87 km^2^ areas have transitioned from informal (or unplanned) built-up (0.09 km^2^), water bodies (1.57 km^2^), green spaces (2.19 km^2^), and vacant areas (175.02 km^2^) into the formal (or planned) urban built-up area. Whereas, overall, 72.88 km^2^ of city area also changed from formal (or planned) built-up (0.76 km^2^), water bodies (0.07 km^2^), green spaces (1.04 km^2^), and vacant areas (71.01 km^2^) into the informal (or unplanned) built-up uses between 2000 and 2020 as specified in Table 6. Simulation model results revealed that most vacant areas have transitioned into planned and unplanned built-up land use land covers (LULC). The transitions from all land use land cover (LULC) areas to formal and informal urban built-up areas between 2000 and 2020 are further outlined in Fig. 7.

**Table 6:** Land use transition from LULC planned and unplanned built-up between 2000 and 2020.

LULC Classes	Planned Built-up (km^2^)	Unplanned Built-up (km^2^)
Planned Built-up	–	0.76
Unplanned Built-up	0.09	–
Water Bodies	1.57	0.07
Green Spaces	2.19	1.04
Vacant	175.02	71.01
Total Area (km^2^)	178.87	72.88

### 4.4 Changes in formal and informal urban built-up growth

Urban built-up growth change rate every five years (i.e., 2000–2005, 2005–2010, 2010–2015, and 2015–2020) between 2000 and 2020 are studied. Previous 20 years of urban development trends considering both planned built-up and unplanned built-up land use land cover change (LULCC) spatiotemporal information are extracted (i.e., 2000–2005, 2005–2010, 2010–2015, and 2015–2020) adopting the Land Change Modeler in IDRISI Selva 17.0. The planned and unplanned urban built-up areas' net change values are calculated in square kilometers.

However, planned urban built-up growth net changes are assessed at 23.21 km^2^, 33.48 km^2^, 55.93 km^2^, and 66.25 km^2^, whereas unplanned urban built-up growth net transition is observed at 17.2 km^2^, 24 km^2^, 15.16 km^2^, and 16.51 km^2^ in between 2000 and 2005, 2000–2010, 2010–2015, and 2015–2020 time periods.

More importantly, the rate of change in planned and unplanned urban built-up growth with five years of intervals (i.e., 2000–2005, 2005–2010, 2010–2015, and 2015–2020) statistics between 2000 and 2020 are evaluated. Spatiotemporal changes in formal and informal urban built-up growth are detected individually every five years. Consequently, planned urban built-up growth is analyzed by 1.23, 1.63, 2.40, and 2.49 average yearly rates. Whereas unplanned urban built-up growth changed by 2.13 %, 2.59 %, 1.51 %, and 1.52 % annually between 2000 and 2005, 2005–2010, 2010–2015 and 2015–2020 intervals is also analyzed.

The rate of change results indicate that the planned urban built-up growth has consistently increased; however, gains in the unplanned urban built-up growth are also alarming as it grew 2.13 % on average per year between 2000 and 2005. Later, a 2.59 % average growth rate was observed between 2005 and 2010 time period. Meanwhile, unplanned urban built-up growth was expanded by 1.51 % annually from 2010 to 2015. Besides, in the more recent interval (2015–2020), the informal built-up growth also increased at 1.52 % on average per annum in Table 7.

**Table 7:** Rate of change in planned and unplanned urban built-up.

LULC Classes	Area Change (km^2^)			
	2000–2005	2005–2010	2010–2015	2015–2020
Planned Built-up	23.21	33.48	55.93	66.25
Unplanned Built-up	17.2	24	15.16	16.51
Total Urban Built-up	40.42	57.48	71.09	82.76
	Average Annual LULC Change Rate (%)			
Planned Built-up	1.23	1.63	2.40	2.49
Unplanned Built-up	2.13	2.59	1.51	1.52
Total Urban Built-up	1.5	1.93	2.13	2.21

### 4.5 Urban developmental trend analysis

The existing developmental trend of Karachi is analyzed through GIS-based spatial data maps. The past 20 years (2000–2020) of land use land cover (LULC) information is extracted using ArcMAP10.8 to find the urban built-up growth trend. High-resolution geo-referenced previous land use land cover (LULC) maps and satellite imagery were taken from metropolitan governmental departments and generated different spatial data maps between 2000 and 2020.

**Fig. 8:**
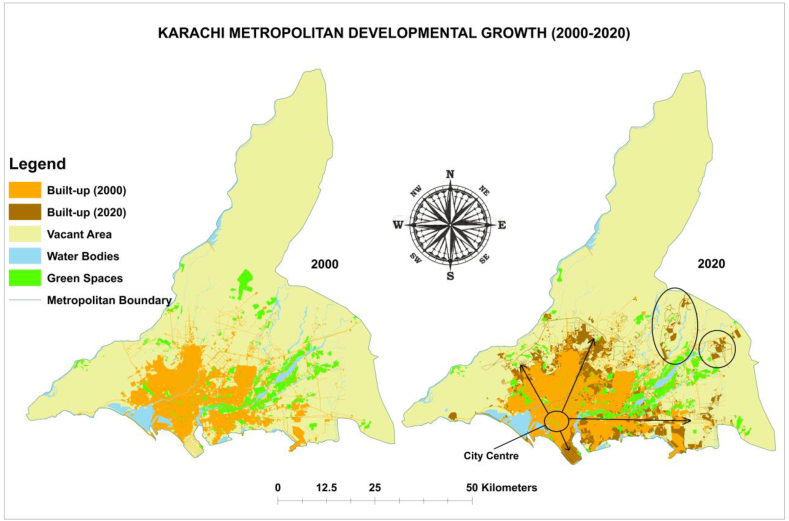
Existing urban built-up growth trend in Karachi (2000–2020) by author).

Having maximum spatial data accuracy, the author digitized the spatiotemporal land use land cover (LULC) maps in ArcGIS 10.8. Resultantly, a total of 498.03 km^2^ of urban built-up growth was observed in 2000, which had mainly grown in Northwest (NW), Northeast (NE), East (E), and West (W) directions. Currently, developmental growth has reached 749.78 km^2^, which is often spreading over Northwest (NW), North (N), North-east (NE), East (E), and Southeast (SE) directions. Additionally, two new satellite towns (i.e., Bahria Town and DHA City) are developed within the sub-urban premises of Karachi in the northeast (NE), as illustrated in Fig. 8.

More importantly, built-up considering formal (or planned) and informal (or unplanned) urban built-up growth change trends are identified based on 20 years between 2000 and 2020. A total of 498.03 km^2^ of urban built-up growth was assessed in 2000, out of which 353.72 km^2^ of formal (or planned) built-up increase was noted and 144.31 km^2^ informal (or unplanned) built-up growth in Karachi. Mostly unplanned built-up had expanded towards the Northwest (NW) direction, and planned built-up grew towards North (N) and Northeast (NE) directions. Moreover, both planned and unplanned built-up, as mixed development was observed towards the East (E) direction.

Simultaneously, an overall 749.78 km^2^ of existing (2020) urban built-up growth is examined, wherein 532.59 km^2^ of planned built-up growth was detected and 217.19 km^2^ of unplanned built-up growth in Karachi. Mainly informal built-up uses have grown towards the Northwest (NW) direction, and formal built-up towards the North (N) and Southeast (SE) directions. Also, both formal and informal built-up, as mixed developments have been spreading over Northeast (NE) and East (E) directions. The inclusive urban built-up growth change trend scenarios are further indicated in Fig. 8.

### 4.6 Informal urban built-up growth prediction

**Fig. 9:**
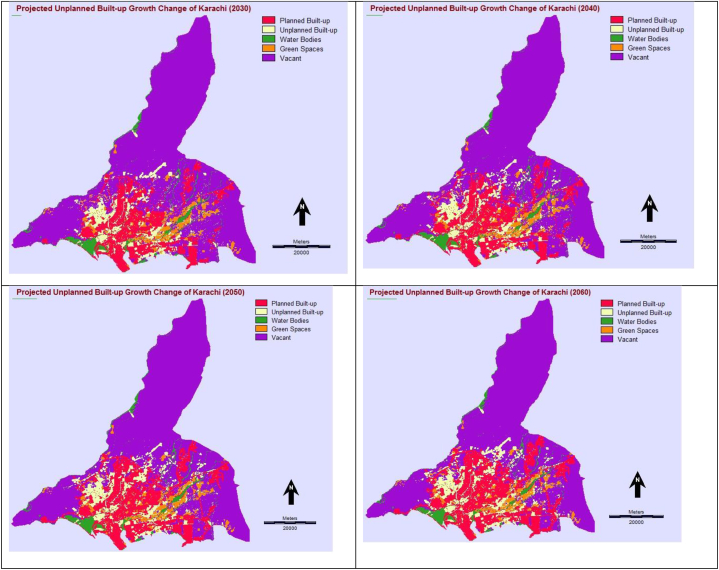
Predicted urban built-up growth 2030, 2040, 2050, 2060.

The future trend of informal (or unplanned) urban built-up growth was predicted for the next 40 years up to 2060 with ten years of interval (i.e., 2030, 2040, 2050, and 2060) using simulation models in IDRISI software. Firstly, four various time interval-based transition probability matrixes were produced using the Markov model, putting transition time periods (20 years) between earlier (2000) and later (2020) land use land cover (LULC) spatial data maps and forward projecting time periods from the later (2020) map (i.e., 10, 20, 30 and 40 years). The transition probability matrixes of various time periods are provided in Table 8, Table 9, Table 10, Table 11. An integrated Cellular Automata Markov (CA-Markov) simulation model was then applied and projected future informal (or unplanned) urban built-up growth into four phases: 2030, 2040, 2050, and 2060, described in Fig. 9.

**Table 8:** Markov chain transition probability matrix up to 2030.

	Planned Built-up	Unplanned Built-up	Water Bodies	Green Spaces	Vacant
Planned Built-up	0.9884	0.0055	0.0000	0.0026	0.0035
Unplanned Built-up	0.0059	0.9802	0.0000	0.0031	0.0108
Water Bodies	0.0077	0.0001	0.9779	0.0041	0.0101
Green Spaces	0.0152	0.0093	0.0011	0.8616	0.1129
Vacant	0.0306	0.0118	0.0004	0.006	0.9512

**Table 9:** Markov chain transition probability matrix up to 2040.

	Planned Built-up	Unplanned Built-up	Water Bodies	Green Spaces	Vacant
Planned Built-up	0.9772	0.0108	0.0000	0.0046	0.0073
Unplanned Built-up	0.0121	0.9611	0.0000	0.0056	0.0212
Water Bodies	0.0156	0.0005	0.9563	0.0074	0.0201
Green Spaces	0.0325	0.0186	0.0020	0.7516	0.1953
Vacant	0.0591	0.0228	0.0008	0.0106	0.9067

**Table 10:** Markov chain transition probability matrix up to 2050.

	Planned Built-up	Unplanned Built-up	Water Bodies	Green Spaces	Vacant
Planned Built-up	0.9663	0.0161	0.0000	0.0066	0.0111
Unplanned Built-up	0.0184	0.9425	0.0000	0.0080	0.0312
Water Bodies	0.0235	0.0010	0.9352	0.0105	0.0297
Green Spaces	0.0496	0.0277	0.0029	0.6489	0.2709
Vacant	0.0864	0.0335	0.0012	0.0148	0.8641

**Table 11:** Markov chain transition probability matrix up to 2060.

	Planned Built-up	Unplanned Built-up	Water Bodies	Green Spaces	Vacant
Planned Built-up	0.9557	0.0213	0.0000	0.0081	0.0149
Unplanned Built-up	0.0249	0.9244	0.0000	0.0099	0.0408
Water Bodies	0.0316	0.0017	0.9146	0.0130	0.0391
Green Spaces	0.0680	0.0367	0.0036	0.5672	0.3245
Vacant	0.1119	0.0435	0.0015	0.0180	0.8251

However, a total of 829.58 km^2^ of urban built-up growth was projected between 2020 and 2030 (10 years of the interval), out of which 596.86 km^2^ was noted planned (or formal) built-up growth and 232.72 km^2^ of unplanned (or informal) built-up growth. Similarly, 933.37 km^2^ of urban built-up growth was predicted between 2020 and 2040 (20 years of interval), in which planned built-up was projected at 671.09 km^2^ and unplanned built-up 262.28 km^2^. Meanwhile, a total of 1033.32 km^2^ of urban built-up growth was calculated between 2020 and 2050 (30 years of interval), wherein 742.56 km^2^ of planned built-up growth was computed and 290.76 km^2^ of unplanned built-up growth. Overall, 1127.24 km^2^ of urban built-up growth was forecasted between 2020 and 2060 (40 years of interval), out of which 809.61 km^2^ of planned built-up growth was simulated and 317.63 km^2^ of unplanned built-up growth in Karachi.

Urban built-up growth projection results revealed that a total of 377.46 km^2^ of built-up areas could be changed from 749.78 km^2^ to 1127.24 km^2^ between 2020 and 2060, whereas informal (or unplanned) built-up areas are also anticipated to be transitioned from 217.19 km^2^ to 317.63 km^2^, about 100.44 km^2^ in next 40 years up to 2060. The predicted various land use land cover (LULC) transitions into urban built-up (planned and unplanned) areas and informal (or unplanned) urban built-up changes are briefly elucidated in Table 12.

**Table 12:** Predicted urban built-up growth change statistics for 2030, 2040, 2050, and 2060.

LULC Classes	2030		2040		2050		2060	
	Area (km^2^)	Area (%)	Area (km^2^)	Area (%)	Area (km^2^)	Area (%)	Area (km^2^)	Area (%)
Planned Built-up	596.86	71.95	671.09	71.90	742.56	71.86	809.61	71.82
Unplanned Built-up	232.72	28.05	262.28	28.10	290.76	28.14	317.63	28.18
Total Urban Built-up Area	829.58	100	933.37	100	1033.32	100	1127.24	100
Built-up	829.58	22.55	933.37	25.37	1033.32	28.09	1127.24	30.64
Water Bodies	132.86	3.61	131.15	3.57	129.46	3.52	127.82	3.47
Green Spaces	157.61	4.28	154.02	4.19	150.42	4.09	147.05	4.00
Vacant	2558.48	69.55	2459.99	66.87	2365.31	64.30	2276.42	61.88
Overall Total Area	3678.53	100	3678.53	100	3678.53	100	3678.53	100

Additionally, knowing the projected future spatiotemporal urban developmental trend for the next 40 years (i.e., 2030, 2040, 2050, and 2060), the CA-Markov outputs were also mapped in IDRISI. More specifically, informal (or unplanned) built-up growth change was analyzed between different periods. Resultantly, most informal urban built-up areas can expand towards the Northwest (NW), North (N), and East (E) directions between 2020 and 2030, whereas informal (or unplanned) urban built-up growth probably may increase further in the Northeast (NE), East (E), West (W), and Northwest (NW) directions in 2040.

Meanwhile, the informal built-up area may grow again towards the Northwest (NW), Northeast (NE), and East (E) directions in 2050. Besides, informal built-up growth could often spread over Northwest (NW), Northeast (NE), and East (E) directions in 2060. The projected informal urban built-up growth scenarios are exhibited in Fig. 9.

## 5 Discussion

This research was focused on urban built-up growth considering informal (or unplanned) built-up change. Hence, urban built-up land use/land cover was segregated into two land uses such as formal (or planned) built-up and informal (or unplanned) built-up areas. The existing trend of informal (or unplanned) urban built-up growth happened for the past 2 decades was then examined in metropolitan Karachi. Similarly, informal (or unplanned) built-up growth change statistics were assessed based on five years of interval (i.e., 2000–2005, 2005–2010, 2010–2015, and 2015–2020) between 2000 and 2020 in metropolitan Karachi.

Results revealed that both formal (or planned) and informal (or unplanned) built-up growth is changing with time, but metropolitan land transition into informal (or unplanned) built-up area condition is alarming, as it has changed from 144.31 km^2^ to 217.19 km^2^ with 72.88 km^2^ since previous 2 decades. Mostly informal (or unplanned) built-up land use/land cover have converted from vacant (71.01 km^2^) LULC and remaining from planned built-up (0.76 km^2^), water bodies (0.07 km^2^), and green spaces (1.04 km^2^) land use/land cover in metropolitan Karachi. Research outcomes concluded that the metropolitan Karachi's built-up land use/land cover has grown with time as it has changed from 498.03 km^2^ (13.54 %) to 749.78 km^2^ (20.38 %) with 251.75 km^2^ spatiotemporal variance in the past 20 years between 2000 and 2020. Mostly vacant land use/land cover has dramatically transitioned into urban built-up LULC in metropolitan Karachi.

The future urban developmental change trend, the urban built-up growth considering informal (or unplanned) built-up growth was also projected for the next 40 years up to 2060 in metropolitan Karachi. Four different interval scenarios of informal (or unplanned) built-up growth in between 2020 and 2030, 2020–2040, 2020–2050, and 2020–2060 were calculated using the integrated CA-Markov model in IDRISI.

### 5.1 1Research implications and limitations

This research would be useful in assisting academia and urban planning/development professionals while observing the past, present, and future trends of urban land conversion mainly into the informal (or unplanned) built-up change using the integrated Cellular Automata Markov (CA-Markov) Model at metropolitan scale urban areas globally, particularly in the developing countries. Therefore, this study could lead urban planners, decision-makers, city administrators, and various stakeholders to recognize the existing conditions of urban built-up growth change and observe the future possibilities of urban land change in Karachi during the city's development process.

This research is conducted focusing on informal (or unplanned) built-up (i.e., sprawl/spontaneous/unplanned development; e.g., slums, katchi bodies, squatter settlements, leap-frog/disperse settlements) growth at the first-tier (or metropolitan scale) city of Pakistan (i.e., a South Asian developing country); however, in future, this type of research could carry out further at second-tier (or secondary) and third-tier (or tertiary) cities of developing countries including Pakistan. Moreover, in this research, the overall informal (or unplanned) built-up growth at the metropolitan scale has been assessed using geo-information system tools. The rate of change of informal (or unplanned) built-up segregating into infill, outskirts, and scattered growth at metropolitan, secondary, and tertiary/small cities could further identify in future observation the most affected areas by informal (or unplanned) built-up change.

## 6 Conclusion

This research analyzed informal (or unplanned) urban built-up growth trends in the rapidly developing Karachi. The informal (or unplanned) built-up growth trending since the past two decades between 2000 and 2020 and its prediction for the next four decades between 2020 and 2060 are analyzed.

The spatial data was obtained and analyzed by adopting various scientific techniques. The maximum accuracy-based spatial data was generated from governmental departments using geo-referenced high-resolution, detailed maps and satellite images of Karachi using ArcGIS10.8. Four land use land cover (LULC) types (i.e., urban/built-up, barren/vacant land, water bodies, and vegetation/green spaces) were categorized considering the Pakistan land classification system [102,117,118].To achieve the research objectives, the built-up land use land cover (LULC) is further segregated into two types: formal (i.e., Planned/proper urban developed or under-developing settlements) and informal (i.e., sprawl/spontaneous/unplanned development, e.g., slums, katchi bodies, squatter settlements, leapfrog/disperse settlements) built-up uses. However, the previous and future trends of informal urban built-up growth were examined using the Land Change Modeler (LCM) and the integrated Cellular Automata Markov (CA-Markov) simulation model in IDRISI software. More significantly, formal (or planned) and informal (or unplanned) built-up growth have also transitioned with time. Still, the transitioning of metropolitan land into informal (or unplanned) built-up situations is alarming.

Presently (2020), Karachi's built-up growth has been spreading over the Northwest (NW), North (N), Northeast (NE), East (E), and Southeast (SE) directions, which was observed towards the Northwest (NW), Northeast (NE), East (E), and West (W) directions 20 years ago in 2000 (i.e., expended between 1986 and 2000 time period). Simultaneously, the existing (2020) informal (or unplanned) urban built-up growth also has been expanded over the Northwest (NW), Northeast (NE), and East (E) directions, respectively, which was noted towards the Northwest (NW) and East (E) directions in 2000.

More notably, informal urban built-up growth could also increase in the future if existing developmental trends continuously occur in Karachi. Informal (or unplanned) built-up growth could spread over in different directions, but more often towards Northwest (NW), East (E), and Northeast (NE) directions. However, it is recommended that Karachi's authorities pay more attention to these directions while making a new Master Plan of Karachi to avoid informal (or unplanned) built-up change in the future.

## Funding statement

This research did not receive any specific grant from funding agencies in the public, commercial, or not-for-profit sectors.

## Additional information

No additional information is available for this paper.

## Data availability statement

Data will be made available on request.

## CRediT authorship contribution statement

**Cai Li:** Writing – original draft, Supervision, Software, Methodology, Investigation, Formal analysis, Data curation, Conceptualization. **Sania Khan:** Writing – review & editing, Writing – original draft, Supervision, Resources, Project administration. **Noman Sahito:** Writing – review & editing, Writing – original draft, Visualization, Validation, Supervision, Software, Resources, Project administration, Methodology, Investigation, Formal analysis, Data curation, Conceptualization. **Muhammad Yousif Mangi:** Writing – original draft, Software, Methodology, Investigation, Formal analysis, Data curation, Conceptualization. **Wadi B. Alonazi:** Writing – review & editing, Resources, Funding acquisition.

## Declaration of Competing interest

The authors declare no conflict of interest.
